# Can Regulation Affect the Solvency of Insurers? New Evidence from European Insurers

**DOI:** 10.1007/s11294-023-09867-w

**Published:** 2023-03-20

**Authors:** Evaggelia Siopi, Thomas Poufinas, James Ming Chen, Charalampos Agiropoulos

**Affiliations:** 1 Department of Economics, Democritus University of Thrace, Komotini, Greece; 2 College of Law, Michigan State University, East Lansing, MI USA; 3 School of Economics, Business and International Studies, University of Piraeus, Piraeus, Greece

**Keywords:** Insurance companies, Solvency, Machine learning, LASSO regression, G17, G22, G28

## Abstract

Successive crises in the early twenty-first century prompted regulators around the world to ask financial institutions to implement a series of regulations. These measures aimed to increase transparency, improve consumer and investor protection, restructure financial capital, stabilize insurance and pension markets, and improve solvency. The Solvency II framework introduced in the European Union applied these principles to insurance companies. This study attempts to predict the solvency of an insurer within a set of European insurers. The dataset consists of 29 insurance groups that operate across the European Union with a country of origin within the European Union for the period 2016 to 2020. The variables were constructed from annual financial statements retrieved from (Thomson Reuters) DataStream. The solvency capital requirement ratios were obtained manually from the solvency financial condition reports of each group. Regularized linear regression applying a ℓ_1_/ least-absolute-shrinkage-and-selection-operator penalty showed that the reinvestment rate, cash and equivalents, long term investment, and losses-benefits-and-adjustments expenses have the greatest predictive impact on the solvency of insurers. The contribution of this paper lies in the identification of determinants that allow insurance companies to maintain strong solvency capital requirement ratios so that they can maintain internal operations with minimal interruption.

## Introduction

Since the financial crisis that began in 2007–2008, the European Union (EU) has undertaken an ambitious scheme for supervisory and regulatory reform. That crisis spurred a severe worldwide economic crisis that marked the next decade and the first quarter of the twenty-first century. Until the coronavirus (COVID) pandemic broke out in 2020, it was probably the most serious financial crisis since the Great Depression.

The 2008 bankruptcy of Lehman Brothers sparked an international banking crisis. The European debt crisis began with a deficit in Greece in late 2009. The 2008–2011 Icelandic financial crisis involved the failure of all three of that country’s major banks. Relative to the size of its economy, the Icelandic crisis was the largest economic collapse suffered by any country in history.

Regulatory authorities reacted. The United States (U.S.) enacted the Dodd-Frank Wall Street Reform and Consumer Protection Act of 2010 in order to "promote the financial stability of the United States" (U.S. Library of Congress, 2010, PL 111–203). In 2009, countries around the world adopted the Basel III capital and liquidity standards. Because regulation is a complex interaction between politicians, civil servants, industry, interested groups, regulatory bodies, and consumers, its true impact demands close scrutiny.

The European Insurance and Occupational Pensions Authority (EIOPA, 2009a, b) introduced new solvency capital requirements as a system of governance and a mechanism for cooperation and coordination between supervisory authorities. Called Solvency II, this scheme took effect on January 1^st^, 2016 and applies to all European insurance and reinsurance companies (EIOPA, 2009a, b, 2015a, b, 2016, 2018). It attempts to create a level playing field for the European insurance sector. In addition to prescribing rules for the governance of insurance companies, Solvency II emphasizes the capital required to cover the assumed risks and safeguard the solvency of insurers.

Solvency II seeks to reduce the risk of failure of an insurance company to cover the claims of the insured and protect policyholders from losses due to such events. In addition, Solvency II sets rules for more detailed, public information included in the Solvency and Financial Condition Report (SFCR) and not just in the Report to Supervisors. Increased transparency should boost confidence in all types of insurance: life insurance, non-life insurance, and reinsurance. As risk taking is the primary component of the business of insurance, an insurer's risk management process is laid out in its own risk and solvency assessment (ORSA) (EIOPA, 2015a, b). ORSA includes a risk-based assessment of the insurer’s solvency needs based on its business profile and own risk appetite. It must be considered in running the business.

This study advances the existing literature in two ways. First, it identifies the most important variables affecting the solvency capital requirement (SCR) ratio, which is vital for the viability of European insurance companies. Second, this study sets a benchmark for monitoring and forecasting the effectiveness of the risk management process that insurers implement. Such forecasting ability is of the utmost importance for the insurance sector, since solvency enables insurers to deliver benefits promised to policyholders and fulfill their social obligations.

## Literature Review

Financial services, banking and insurance have benefited from real-world applications of machine learning. Examples include customer/market segmentation, portfolio optimization, tracking and prevention of money laundering and other illegal financial activities, implementation of smarter and more effective risk management and regulatory compliance in finance and accounting. These capabilities enable organizations to achieve and maintain a long-term competitive advantage (Paltrinieri et al., 2019; Sen & Mehtab, 2021; Lei et al., 2020; Dornadula & Geetha, 2019; Eling et al., 2022; Yu et al., 2021; Leo et al., 2019; Gu et al., 2020; Ye & Zhang, 2019; Zand et al., 2020). The existing literature addresses a wide range of machine learning applications in insurance. These include the prediction of insolvency, fraud detection (in property and casualty insurance), claims (in export credit insurance), customer-risk level, and losses (in property and casualty insurance), claims analysis (in health and travel insurance), lapse-risk management, portfolio insurance strategies, and motor insurance analysis (Table 1).

**Table 1:** Literature review on the applications of ML in the insurance industry

Authors	Year	Application	Techniques used
*Insolvency prediction*			
Rustam and Saragih	(2021)	Insolvency Prediction of Insurance Companies	Random Forest
Kartasheva and Traskin	(2011)	Insurers' Insolvency Prediction	Random Forest
Díaz et al.	(2005)	Prediction of Insolvency of Spanish Non-life Insurance Companies	Two non-parametric ML techniques (See5 and Rough Set), multilayer perceptron, linear discriminant analysis and logistic regression. The ML techniques post higher performance, more understandable and interpretable decision models
Salcedo-Sanz et al.	(2005)	Insolvency Prediction	Simulated annealing (SA) and Walsh analysis for feature selection using SVM as an underlying classifier
*Fraud prediction*			
Severino and Peng	(2021)	Fraud prediction in property insurance based on real-world data from a major Brazilian insurance company	Random forest, gradient boosting, deep neural networks and logistic regression. Ensemble-based methods yielded the best results, with superior average performance compared to the other classifiers
Subudhi and Panigrahi	(2018)	Auto-Insurance Fraud Detection	Univariate, L1 based & tree-based feature selection and decision tree, naïve Bayes, KNN & random forest classification algorithms
Patil and Godbole	(2018)	Fraud Detection	Supervised, unsupervised and hybrid- bagging, boosting, stacking & ensemble learners
Wang and Xu	(2018)	Auto-Insurance Fraud Detection	Deep learning model with latent dirichlet allocation (LDA) based text analytics
*Claim prediction*			
Bärtl and Krummaker	(2020)	Claim Prediction in Export Credit Finance	Decision tree, random forest, neural networks (NN) & probabilistic neural networks (PNN) for prediction and accuracy, Cohen’s K & R^2^ for assessment
Quan and Valdez	(2018)	Predictive Analytics of Claims	Decision trees and their extensions as predictive models for insurance claims, comparing the predictive performance of various univariate tree-based models against multivariate tree-based models
*Risk and loss prediction*			
Mauritsius et al.	(2020)	Risk level predictions of potential customers of the largest life insurance company in Indonesia	The support vector machine (SVM) algorithm, naïve Bayes algorithm, and random forest (RF) were evaluated according to accuracy, recall (sensitivity), and precision. Random forest showed the highest precision, accuracy, and sensitivity
Ding et al.	(2020)	Insurance losses prediction of U.S.-based property and casualty insurance companies	Four popular ML algorithms (linear regression, random forest, gradient boosting machine, and artificial neural networks) were used. Random forest showed the best accuracy and prediction
*Lapses*			
Loisel et al.	(2021)	Lapse risk management	Extreme gradient boosting and support vector machine used to predict whether a policyholder will lapse her/his policy
*Portfolio Insurance*			
Dehghanpour and Esfahanipour	(2018)	Portfolio Insurance Strategy	Adaptive neuro-fuzzy inference systems (ANFIS) for prediction combined with the Markowitz portfolio optimization model for determining optimal portfolio weights
*Other insurance-related analysis*			
Rawat et al.	(2021)	Claim Analysis Two case studies were considered: (i) the health insurance sector, from the perspective of the beneficiary; and (ii) the travel insurance sector, from the perspective of the insurer	Logistic regression, random forest, decision tree, support vector machine, Gaussian naïve Bayes, Bernoulli baïve Bayes, mixed naïve Bayes, K-nearest neighbors. For both case studies random forest was the best classifier with suitable feature selection methods
Kang and Song	(2018)	Aggregate Auto-Insurance Data Analysis	Feature selection techniques to classify the dataset into homogenous risk groups
Rao and Pandey	(2013)	Factors influencing Claims in General Insurance, India	Regression analysis
Guelman	(2012)	Insurance Lost Cost Modelling	Gradient boosting compared to the linear model approach

This study addresses a gap in the literature, the identification of the most important factors affecting SCR ratios. This paper studies the internal (firm-related) factors that allowed insurance companies to maintain SCR ratios that ensure solvency. These factors relate to premiums generated, insurers’ reserves, effectiveness in reinvesting in profitable assets, cash or cash equivalents held, long-term investments, losses and expenses (e.g., management, administrative), size, and income generated by each insurer’s total activity.

## Data and Variables

The dataset consists of 29 insurance groups that operate across the EU, with a country of origin within the EU, from 2016 to 2020. The proxy employed for solvency is the SCR ratio, which is the sum of eligible own funds divided by the SCR, calculated on a consolidated basis. The SCR is the amount of assets that insurance and reinsurance companies are required to hold in order to attain 99.5% confidence that they will be able to meet the claims of policyholders under extreme expected losses. The SCR accounts for life insurance, health insurance, market, credit, operational and counterparty risk and must be recalculated at least once a year.

Eligible own funds are the component of actual own funds that qualify for coverage of the SCR and the minimum capital requirement (MCR), the minimum safety net of capital adequacy over one year. Eligibility is decided by the regulator, includes restrictions on the amount of each tier of capital an insurer can use to cover its SCR and MCR, and must be over 100% (EIOPA, 2020a, b).

The variables were constructed from annual financial statements retrieved from (Thomson Reuters) DataStream (2012). SCR ratios were obtained manually from the SFCR of each group. The variables are defined in Table 2.

**Table 2:** Brief definition and role of the model variables

Target variable		Role
Solvency Capital Requirement (SCR) ratio	Ratio of capital available to support SCR to the SCR	Measures Solvency
Features (Explanatory Variables)		Role
Premiums Earned—Change %	Year-to-year change	Monitors performance and ability to support controlled growth from capital generation
Reserves—Change %	Year-to-year change	Indicates the financial obligations stemming from the policies issued by an insurer
Reinvestment Rate	Per cent	Return received after reinvesting the cash flows of the investments
% Long-Term Debt to Total Capital	Long-term debt as % of total available capital	Reflects the risk borne by an investor as the higher the debt the greater the insolvency risk
Cash and Equivalents	% of Total Assets	Shows emergency funding needs and investment opportunities
Long Term Investments	% of Total Assets	Captures the long-term assets insurers hold to match their long-term liabilities
Total Long-Term Debt	% of Total Liabilities & Shareholders' Equity	Unveils an insurer’s health via asset liquidation to pay off long-term debt
Total Premiums Earned	% of Total Revenue	Compares premium—the principal source of revenue with total revenue
Net Investment Income	% of Total Revenue	Compares the investment outcome with the total revenue
Losses, Benefits, and Net Investment Adjustments Expenses	% of Total Revenue	Compares claim investigation & settlement and underwriting expenses to total revenue
Selling/General/Admin. Expenses	% of Total Revenue	Compares management costs to the total revenue

## Methods

Traditional econometric approaches typically specify a model to be fitted. The model is usually based on economic theory and specifies a fixed functional form that includes a dependent variable and one or more independent variables. The ordinary least squares (OLS) procedure is the most common method in general and seeks to minimize the sum of the squared residuals. Given a regression line through the data, the sum of the squared residuals is estimated as the sum of the squares of the distances of the data points from the regression line. In contrast, machine-learning (ML) approaches capture data patterns and apply them to a wide range of problems. ML techniques are efficient and accurate in prediction and classification (Berry & Linoff, 2004; Kudyba, 2014; Sarker, 2021; Thompson, 2014). ML is primarily concerned with prediction: producing the best predictions of *y* given available data *X*. Informally, “machine learning belongs in the part of the toolbox marked $\widehat{y}$ rather than in the more familiar $\widehat{\beta }$ component” (Mullainathan & Spiess, 2017, p. 88). ML methods attempt to find generalizable patterns in the available data and exploit those patterns to make accurate predictions.

Since the objective of ML is to make accurate predictions, ML methods must be evaluated differently than econometric methods. The latter are commonly evaluated using metrics that are calculated using in-sample tests (e.g. *R*^2^, p-values) and out-of-sample tests (e.g., bias, accuracy). As in econometrics, ML methods typically partition the data into training and testing data (in-sample and out-of-sample, respectively). Holdout testing data are used to evaluate the model that has been fitted using the training data. ML methods typically employ cross-validation to train a model. This analytical framework follows the approach introduced by Chen (2021).

## Findings

### Summary statistics-Correlation analysis

Summary statistics of the key variables (Table 3) report negative values in the annual change of insurance premiums and reserves, as well as the reinvestment ratio and net investment income. Furthermore, some companies exhibit zero long-term debt. There is large variation in the exposure to long-term investments from 14.34% to 90.62% of assets. Expenses also exhibit great variation. Losses, benefits and adjustments expenses ranged from 22.46% to 131.26%. Selling, general and administrative expenses ranged from 0.04% to 38.13% of total revenue. In terms of solvency, one company in 2020 fell below the 100% security threshold and posted a SCR ratio of 66%.

**Table 3:** Summary statistics of the model variables for 2016–2020

	Mean	Standard deviation	Min	Max
Premiums Earned—Change %	0.0182	0.0802	-0.4230	0.2180
Reserves—Change %	0.0273	0.1139	-0.2650	0.9820
Reinvestment Rate	0.0396	0.0588	-0.2150	0.3290
% Long-Term Debt to Total Capital	0.2206	0.1449	0.0000	0.7790
Cash and Equivalents	0.0423	0.0570	0.0020	0.3019
Long-Term Investments	0.6490	0.1764	0.1434	0.9062
Total Long-Term Debt	0.0428	0.0519	0.0000	0.4792
Total Premiums Earned	0.8713	0.1547	0.3615	1.6126
Net Investment Income	0.1062	0.1245	-0.6170	0.5754
Losses, Benefits, and Adjustments Expenses	0.6474	0.1694	0.2246	1.3126
Selling/General/Admin. Expenses	0.1037	0.0825	0.0004	0.3813
SCR ratio	2.0281	0.3922	0.6600	3.4100

Source: Authors’ estimates using Python and annual financial statements from the Thomson Reuters DataStream (2012) database provided by Refinitiv. The SCR ratios were obtained manually from the SFCR of each insurance firm. *N* = 145

The SCR ratio has a positive correlation with the annual change in insurance premiums and reserves, the reinvestment ratio, net investment income, long-term debt and expenses in losses, benefits, and adjustments. In contrast, it has a negative correlation with cash and equivalents, premium and selling, and general and administrative expenses (Table 4).

**Table 4:** Correlation matrix of the model explanatory variables for 2016–2020

	Premiums earned—change %	Reserves—change %	Reinvestment rate	% LT debt to total capital	Cash and equivalents	Long-term investments	Total long-term debt	Total premiums earned	Net investment income	Losses, benefits, and adjustments expenses	Selling/General/Admin. Expenses	Solvency Capital Requirement (SCR) ratio
Premiums Earned—Change %	1.000											
Reserves—Change %	0.254	1.000										
Reinvestment Rate	0.067	0.059	1.000									
% LT Debt to Total Capital	-0.088	-0.040	-0.091	1.000								
Cash and Equivalents	0.033	0.003	-0.136	-0.519	1.000							
Long-Term Investments	0.081	0.161	0.309	0.223	-0.646	1.000						
Total Long-Term Debt	-0.002	0.005	-0.029	0.818	-0.302	0.165	1.000					
Total Premiums Earned	0.082	-0.061	-0.084	-0.220	0.234	-0.155	-0.017	1.000				
Net Investment Income	-0.087	0.161	0.079	0.322	-0.283	0.327	0.172	-0.749	1.000			
Losses, Benefits, and Adjustments Expenses	-0.038	-0.162	-0.010	0.130	-0.367	0.199	-0.119	0.015	-0.164	1.000		
Selling/General/Admin. Expenses	0.004	0.051	-0.036	-0.356	0.257	-0.120	-0.359	0.073	-0.162	-0.364	1.000	
SCR ratio	0.051	0.007	0.190	0.110	-0.386	0.425	0.056	-0.102	0.172	0.204	-0.081	1.000

Source: Authors’ estimates using Python and annual financial statements from the Thomson Reuters DataStream (2012) database provided by Refinitiv. The SCR ratios were obtained manually from the SFCR of each insurance firm. The number of observations for each variable is 145

### Results

The estimation results of all regression and supervised ML models showed that our model is weakly predictive, Pooled OLS, random forest, extra trees, eXtreme gradient boosting (XGBoost), gradient boosting, AdaBoost, support vector regression (SVR) and multi-layer perceptron (MLP), struggled to find just the right combination of independent variables to make good predictions. Traditional linear regression did not exceed 0.30 in R^2^ (Table 3). The metrics used to analyze regression models are R^2^ and the root mean squared error (RMSE) (Table 5).

**Table 5:** Outcome of the eight regression models for the period 2016–2020

	R^2^		RMSE	
	Train	Test	Train	Test
Linear Regression	0.247743	0.02163	0.867327	1.052815
Random Forest Regression	0.895306	0.396792	0.323564	0.826673
Extra Trees Regression	0.968077	0.470771	0.178671	0.774323
XGB Regressor	0.895214	0.241988	0.323707	0.926698
Gradient Boosting Regressor	0.579085	0.335906	0.64878	0.867392
AdaBoost Regressor	0.82527	0.298078	0.418008	0.891753
SVR	0.798679	0.197611	0.448688	0.953439
MLP Regressor	0.290617	0.250762	0.842249	0.921319
No of Observations	108	37	108	37

Source: Authors’ estimates using Python and annual financial statements from the Thomson Reuters DataStream (2012) database provided by Refinitiv. The SCR ratios were obtained manually from the SFCR of each insurance firm

### Implementation of Regularized linear models-LASSO

A modification of linear regression is the least absolute shrinkage and selection operator (LASSO). The loss function in LASSO is changed to reduce the model's complexity by limiting the sum of the absolute values of the model coefficients:

1 $$Loss function=OLS+a*summation$$

where the summation is the absolute value of the magnitude coefficients.

**Fig. 1:**
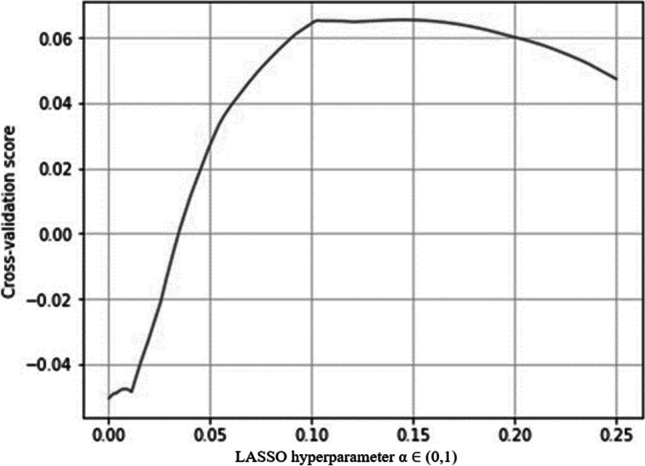
LASSO hyperparameter α reaches its optimal value at 0.1452999999999999998. Source: Authors’ estimates using Python and annual financial statements from the Thomson Reuters DataStream (2012) database provided by Refinitiv for 2016–2020. The SCR ratios were obtained manually from the SFCR of each insurance firm

The default value of the regularization parameter in LASSO regression (given by *α*) is 1, where $a$ is the parameter that balances the amount of emphasis given to minimizing the residual sum of squares (RSS) versus minimizing the sum of squares of coefficients. At *α* = 0, LASSO is equivalent to OLS. RSS is the sum of the squared errors between the predicted and actual values in the training data set. The larger the value of $\alpha$, the more aggressive the penalization. The LASSO hyperparameter $a$ reached its optimal value at 0.1453 (Fig. 1).

LASSO has the effect of reducing coefficients to zero if they do not contribute significant predictive value. The sparsity induced by LASSO indicates significance, akin to the role of *p*-values informal statistics.

### LASSO model selection using an information criterion: AIC or BIC

**Fig. 2:**
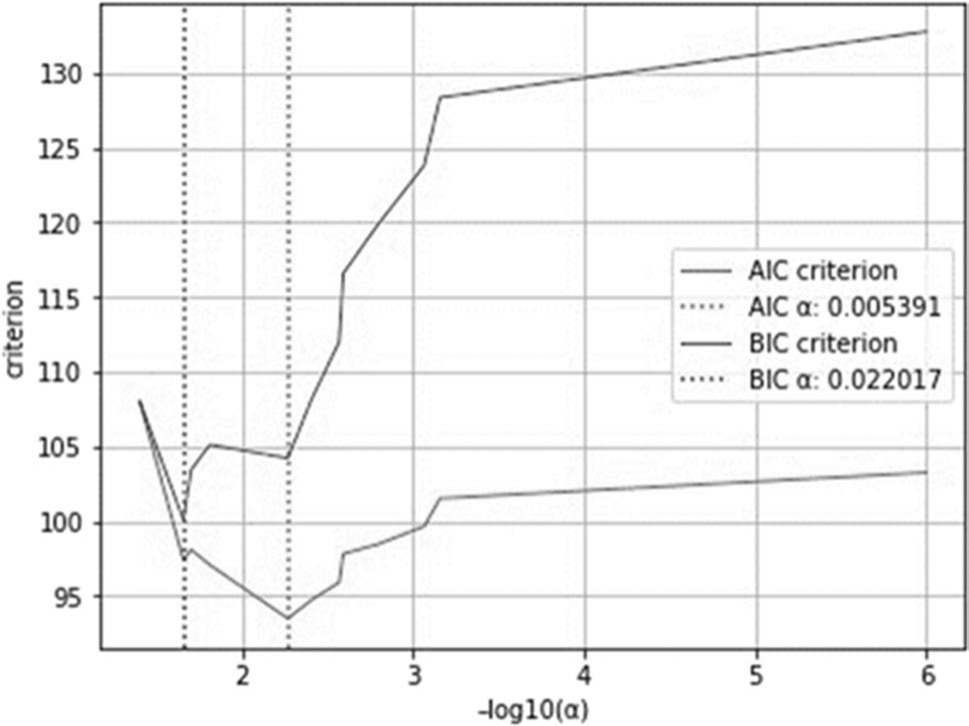
Selecting the LASSO hyperparameter *α* via AIC and BIC. Source: Authors’ estimates using Python and annual financial statements from the Thomson Reuters DataStream (2012) database provided by Refinitiv for 2016–2020. The SCR ratios were obtained manually from the SFCR of each insurance firm. Due to tiny values, they are plotted on a negative base-10 logarithmic scale

The Akaike information criterion (AIC) or the Bayes information criterion (BIC) was used to select the optimal value of the regularization parameter $a$. Before fitting the model, the data were standardized. The AIC and BIC values can be plotted for different values of $a$. The vertical lines in the plot correspond to the $a$ chosen for each criterion. The selected $a$(Fig. 2) corresponds to the minimum of the AIC and BIC criterion (Pedregosa et al., 2011).

As α increases toward its optimized value of 0.1453, LASSO turns more coefficients into zero. The reinvestment rate, cash and equivalents, long-term investment, losses-benefits-and-adjustments expenses were selected from the LASSO regression. LASSO results are best understood through a comparison with the results of conventional OLS regression, which indicates that only the losses-benefits-and-adjustments expenses variable is statistically significant (Table 6). The coefficients of all four variables post the same sign in both regressions though. The other six explanatory variables were assigned zero coefficients at a relatively aggressive value of the LASSO complexity parameter *α*. In effect, LASSO regression reduced the dimensionality of the model from 10 to 4.

**Table 6:** LASSO regression results compared with OLS results for the period 2016–2020

Features	OLS Coefficients	OLS p-values	LASSO Coefficients	LASSO p-values
Premiums Earned—Chg %	0.026873	0.771	0	1.000
Reserves—Chg %	-0.015043	0.876	0	1.000
Reinvestment Rate	0.13127	0.169	0.012228**	0.009
% LT Debt to Total Capital	-0.286764	0.168	0	1.000
Cash & Equivalents	-0.160224	0.277	-0.033657**	0.008
Long Term Investments	0.218395	0.100	0.230712***	0.001
Total Long-Term Debt	0.316752	0.105	0	1.000
Total Premiums Earned	0.032128	0.829	0	1.000
Net Investment Income	0.097143	0.552	0	1.000
Losses, Benefits, and Adjustments Expenses	0.285086	0.025702*	0.044272**	0.007
Selling/General/Admin. Expenses	0.169214	0.132	0	1.000

Statistical significance: *** *p* ≤ 0.001;** 0.01; * 0.05; + 0.10

Source: Authors’ estimates using Python and annual financial statements from the Thomson Reuters DataStream (2012) database provided by Refinitiv. *N* = 145

LASSO regression based on standardized data allows the resulting beta coefficients to be read directly. Although Table 6 reports a corresponding vector of *p*-values, the sparsity induced by this regression method is unequivocally clear and decisive. The zero-coefficient trick replaces or complements the more conventional removal of variables with a high (non-significant) *p-*value.

In this study, the absolute value of the long-term investment coefficient exceeds the sum of the absolute value of the other three non-zero coefficients. Therefore, that variable commanded an overwhelming share of coefficient importance (defined as the absolute value of each non-zero coefficient divided by the sum of the absolute value of all non-zero coefficients). Therefore, LASSO reports the subset of predictive variables within the ℓ_0_ quasi-norm of variables with non-zero coefficients.

Finally, the negative sign attached to the coefficient for cash and equivalents should be highlighted. This is the only negative variable in the new ℓ_0_ vector of coefficients. Cash is not a risk-free asset, especially with respect to the solvency of financial institutions. Cash earns so little return that it undermines preparedness for future crises (Danielsson et al., 2016).

## Discussion

The LASSO regression showed that the reinvestment rate, cash and equivalents, long term investment, and losses-benefits-and-adjustments expenses can predict the solvency of insurance companies during the period under investigation. Insurance companies operated under a low-interest rate environment and continue to earn less investment income. Annual investment returns are reinvested to generate additional future returns.

However, reinvestment at lower yields has a measurable impact on an insurer’s future financial health. Older, higher-yielding, maturing securities and cash are reinvested at current (lower) market rates, leading to reduced investment income. As a result, insurance companies must either hold more assets in the future to earn the same investment income, or else hold riskier assets to achieve better returns. The reinvestment rate can be considered as a tool for risk management, which discourages insurers from investing in risky portfolios and endangering their solvency ratio. Risk-averse insurers want to avoid losses from risky investments, even though they may benefit in the short-term.

Insurance companies are long-term investors. They invest premiums paid by policyholders. Due to the long-term nature of many products (such as annuities and life insurance policies), insurers invest in long-term assets to match their long-term liabilities. However, under Solvency II, assets and liabilities are valued mark-to-market. Consequently, short-term market movements pose a risk that must be managed. Mark-to-market valuation ensures that the SCR ratios reflect an insurer’s true economic position. Therefore, mark-to-market valuation is an instrument for risk management and policyholder protection, even though it does not fully capture the investments’ long-term horizon.

The solvency capital requirements motivate insurers to match the duration of their assets and liabilities. The better the duration match, the lower the solvency capital requirement is. SCR ratios increase insurers' appetite for long-term assets. Insurers are free to make prudent investments, and capital requirements will depend on the actual risk associated with those investments.

Cash is not a risk-free asset. There are differences between the SCR needed to cover cash deposits at a bank and other cash equivalents. Solvency II assumes that the loss (given default) for cash at a bank is 100%. The EIOPA estimates that approximately €190 billion in cash and cash equivalents were held on the balance sheets of European insurers at the end of the second quarter of 2020. A Euribor of -0.6% implies that roughly €1 billion of this cash will be lost through negative yields over the next 12 months (EIOPA, 2020a, b).

Losses-benefits-and-adjustments expenses reflect the cost of investigating and settling insurance claims, relative to an insurance company’s gross revenue. Investigations are necessary to prevent fraud and reduce exaggerated claims; in essence, to verify the amount of the loss. The business of insurance requires fair and prompt payment of valid claims. When an insurance company refuses claims without adequate investigation and fails to pay promptly and fairly when liability is clear, many insureds may sue to recover underpayments. If an insurance company loses many underpayment lawsuits, such defeats indicate that the insurance company is routinely underpaying claims. Therefore, losses-benefits-and-adjustments expenses can provide early warning of systematic under payment relative to gross revenue.

## Conclusion

Five years after the implementation of the Solvency II Directive, this study makes two primary contributions. First, it identifies the most important factors in predicting SCR ratios and evaluates the impact of these factors on solvency. The reinvestment rate, cash and equivalents, and long-term investments (as part of total assets) and losses-benefits-and-adjustments expenses (as part of total revenue) can be used as benchmarks for monitoring and forecasting SCR ratios. Second, this study attains these results through computational extensions of OLS regression. It makes particularly illuminating use of LASSO.
